# A workflow for deriving chemical entities from crystallographic data and its application to the Crystallography Open Database

**DOI:** 10.1186/s13321-023-00780-2

**Published:** 2023-12-19

**Authors:** Antanas Vaitkus, Andrius Merkys, Thomas Sander, Miguel Quirós, Paul A. Thiessen, Evan E. Bolton, Saulius Gražulis

**Affiliations:** 1 Section of Crystallography and Chemical Informatics, Institute of Biotechnology, Life Sciences Center, Vilnius University, Saulėtekio al. 7, Vilnius, LT-10257 Lithuania; 2 Scientific Computing Drug Discovery, Idorsia Pharmaceuticals Ltd., Hegenheimermattweg 89, Allschwil, 4123 Switzerland; 3 Departamento de Química Inorgánica, Universidad de Granada, Granada, 18071 Spain; 4 National Center for Biotechnology Information, National Library of Medicine, National Institutes of Health, Bethesda, MD 20894 USA; 5 Faculty of Mathematics and Informatics, Vilnius University, Naugarduko g. 24, Vilnius, LT-03225 Lithuania

**Keywords:** Crystallography Open Database, PubChem, Molecular perception, Chemical structure assignment

## Abstract

Knowledge about the 3-dimensional structure, orientation and interaction of chemical compounds is important in many areas of science and technology. X-ray crystallography is one of the experimental techniques capable of providing a large amount of structural information for a given compound, and it is widely used for characterisation of organic and metal-organic molecules. The method provides precise 3D coordinates of atoms inside crystals, however, it does not directly deliver information about certain chemical characteristics such as bond orders, delocalization, charges, lone electron pairs or lone electrons. These aspects of a molecular model have to be derived from crystallographic data using refined information about interatomic distances and atom types as well as employing general chemical knowledge. This publication describes a curated automatic pipeline for the derivation of chemical attributes of molecules from crystallographic models. The method is applied to build a catalogue of chemical entities in an open-access crystallographic database, the Crystallography Open Database (COD). The catalogue of such chemical entities is provided openly as a derived database. The content of this catalogue and the problems arising in the fully automated pipeline are discussed, along with the possibilities to introduce manual data curation into the process.

**Supplementary Information:**

The online version contains supplementary material available at 10.1186/s13321-023-00780-2.

## Introduction

The knowledge about how a molecular structure arranges itself in 3-dimensional space is essential for chemistry, drug design, *in silico* docking, crystal structure prediction, as well as for calculating molecular and material properties. Several ways exist to obtain information on molecular structure which simultaneously encompasses chemical entity information (bond types and orders, resonance forms, unpaired electrons, and lone electron pairs), physical properties (atom charges and molecular surfaces) and geometric configuration (exact placement of atoms in 3D space relative to each other). One way is to start with a molecular graph taken from an MDL Molfile or SMILES encoding and to use force fields to obtain atomic coordinates [1] of the molecule using energy minimization techniques. This type of method can be applied to many chemically synthesized or theoretically conjectured molecules. The drawback of this method, however, is that the reliability of the final 3D coordinates heavily depends on the quality of the force field used and on the assumptions that were made about the molecular properties such as electron delocalization. Consequently, predictions based on force field calculations should preferably be experimentally verified. When it comes to verification of molecular geometry, most often the 3D structure of a molecule is determined using X-ray crystallography.

Molecular properties that depend on exact atom positions can also be investigated using an alternative approach. One can start from known 3D atom positions, preferably determined using an experimental technique such as X-ray crystallography, and work from there to determine the chemical nature of bonds and other atom properties not immediately provided by the experiments. This type of approach has the advantage of ensuring valid 3D atom coordinates in the final result and is therefore taken in the present work. The challenge here is to derive accurate chemical information automatically from the atomic coordinates and atom types alone with the minimal number of additional assumptions.

Multiple methods for the automatic assignment of atomic bonding, bond types and atom charges have been proposed over the years [2–11] with greater focus being placed on organic molecules. Furthermore, the actual implementations of the described algorithms are often not openly available thus complicating their reuse or modification. As a result, these approaches usually cannot be directly applied to small-molecule crystal structures with inorganic or organometallic components. While one previously published work [12] does thoroughly address the chemical diversity observed in small-molecule crystal structures, the presented method still lacks an openly available implementation and relies on statistics derived from a proprietary dataset thus further limiting its use. Moreover, there does not seem to be a clear consensus on how certain compounds such as metallocenes or even more conventional metal-coordination complexes should be represented using simple two-centre bonds [13–15]. Finally, most of the generalized bond and charge assignment algorithms tend to ignore certain information present in crystallographic data files, such as the number of attached hydrogen atoms, even though it may lead to a more correct molecular description.

To address these issues, we have developed a heuristics-based open-source program called cif-perceive-chemistry [16] capable of producing molecular descriptions of stoichiometrically correct small-molecule crystal structures. The program was used to process the entirety of the Crystallography Open Database (COD [17, 18]) resulting in an open-access set of molecular descriptions with experimentally determined atomic coordinates derived from peer-reviewed crystallographic data. Unlike proprietary databases with similar content that may restrict the dissemination of derivative works, the 3-dimensional structures derived from the COD are distributed under the CC0 license, which allows them to be copied freely and used for any purpose such as training machine learning models, identifying materials, docking, or molecular property prediction. The dataset is periodically updated to include the newly deposited COD entries as well as to propagate any significant changes made to the older entries. Instructions on how to retrieve the dataset are provided in Additional file 1: Section S7.

## Methods

**Fig. 1:**
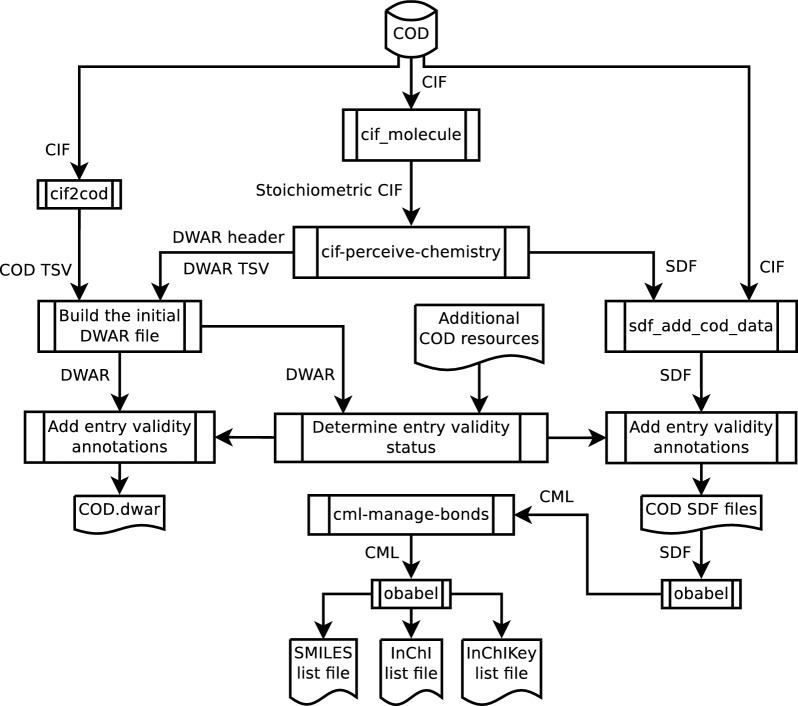
Schema of the workflow used to derive chemical descriptions from crystallographic data in the COD. A more detailed description of the additional COD resources used in entry validity determination is provided in Additional file 1: Section S1

The main steps of deriving chemical descriptions from crystallographic data that were used in this work are shown in Fig. 1. The workflow starts with the restoration of a *stoichiometrically correct molecular ensemble* defined as a minimal grouping of all molecular entities from a single crystal structure with a stoichiometrically correct ratio. This step is followed by the assignment of a chemical structure to the restored stoichiometrically correct molecular ensemble based on the molecular geometry. The derived chemical description dataset is then validated, filtered based on various quality criteria, and enhanced with additional metadata. Finally, the dataset is made more readily accessible and searchable by expressing it in various cheminformatics data formats as well as generating chemical identifiers.

### Crystallographic and chemical data in the COD

The COD is the largest open-access and FAIR collection of small-molecule crystallographic data with the goal of collecting all published crystal structures including those of organic, inorganic, metal-organic compounds, and minerals. Most data in the COD are collected from the supplementary material of peer-reviewed publications while some are also uploaded by crystallographers as personal communications. The COD uses the Crystallography Information File (CIF [19, 20]) as the carrier format with each crystal structure being placed in a separate file and assigned an immutable unique identifier called a COD ID. Descriptions of chemical structures in the COD CIF files are normally limited to the atomic coordinates and atom chemical symbols although some entries do contain additional information such as chemical names, atomic connectivity and oxidation states. There is also an ongoing effort to annotate the entirety of the COD with high-quality manually curated SMILES strings [13], which currently covers slightly more than 40% of all entries.

All changes to the COD files are tracked and recorded using the Subversion version control system, therefore it is possible to refer to a specific version of the entire dataset as well as individual files by providing a revision number. Statements about the COD provided in this work are based on COD revision 265250.

### Restoration of stoichiometrically correct molecular ensembles

A typical CIF file describes a crystal structure in a compact way by recording the structure of an asymmetric unit and a set of crystallographic symmetry operations that can be used to restore the complete unit cell. Under this approach, high symmetry molecular entities are represented only by a subset of their atoms with covalent bonds often being implied by interatomic distances rather than explicitly specified. Restoring a full set of atoms that constitute a stoichiometrically correct molecular ensemble from such representation requires the application of an algorithm that can generate symmetry-equivalent atoms, infer chemical bonds and trace the produced networks of covalently connected atoms.

In this study, a previously published molecular reconstruction algorithm [21, 22] is used as implemented in the cif_molecule program from the cod-tools software package version 3.6.0 [23]. The cif_molecule program takes a conventional CIF file as input and outputs a CIF file that explicitly lists all atoms of a stoichiometrically correct molecular ensemble. In the scope of this work, the output CIF files are also referred to as *stoichiometric CIF files* to clearly differentiate them from their input counterparts and to highlight the semantic differences that were introduced as a result of the molecular reconstruction (see Additional file 1: Section 6.1). Stoichiometric CIF files in the chemical description derivation pipeline are generated by invoking the cif_molecule program with the “--one-datablock-output”, “--preserve-stoichiometry”, “--largest-molecule-only” and “--split-disorder-groups” options which ensure that all molecular entities from the same crystal appear in a single data block with a stoichiometrically correct ratio and with the positional crystallographic disorder resolved to a representative conformation based on the rules described in Additional file 1: Section S2. Crystal structures with compositional disorder, however, are not resolved in this way due to reasons discussed in the “Handling of crystallographically disordered structures” section.

The cif_molecule invocation also includes several additional options responsible for the efficient handling of polymers. In the scope of this work a polymer is defined as a molecular entity which consists of periodically repeating identical subunits and spans an infinite number of crystal unit cells. While cif_molecule can identify polymers and represent them as partial molecular entities of finite size, such descriptions are not yet supported by the chemical comprehension software employed in this pipeline. Due to this, it is sufficient to invoke cif_molecule with the “--max-polymer-span=0” and “--max-polymer-atoms=400” options that retain the polymer detection functionality while also reducing the computational costs by limiting the maximum size of the generated partial molecular entities. Stoichiometric CIF files that contain polymer descriptions are explicitly marked using the _cod_molecule_is_polymer data item and eventually completely excluded from calculations at the later stages of the pipeline.

The cif_molecule program identifies molecular entities as distinct based on their crystallographic independence rather than their chemical structure. This means that molecular entities are considered identical if and only if they are symmetry equivalents of each other (one is mapped to the other by crystal symmetry operations); otherwise, molecular entities are considered different even if their chemical connectivity and other chemical features are the same. As a result, the produced stoichiometric CIF files may sometimes contain multiple instances of the stoichiometrically correct molecular ensemble. This only happens if the input file contains several crystallographically independent copies of the same molecular entity or its fragments in the asymmetric unit. In such cases, all independent molecular entities are restored and treated as distinct since they exist in different physical environments and can potentially have different geometries. For example, they can represent different 3D conformers of the same chemically identical molecular entity. The cif_molecule program does not try to identify whether these conformers should be treated as identical and explicitly delegates this decision to external tools.

Similarly, processing of racemic crystal structures usually results in only one of the enantiomers being retained unless both enantiomorphs are explicitly described in the asymmetric unit as crystallographically independent molecular entities. Though not explicitly marked in any way, these compact racemate descriptions can be easily distinguished from cases which genuinely only describe one of the enantiomers by checking if the molecule crystallised in a non-Sohncke-type space group that is only compatible with achiral crystal structures [24]. Due to this, stoichiometric CIF files output by cif_molecule record the space group number of the input crystal structure so it is readily available for further applications.

Finally, it should be noted that while the main algorithm of cif_molecule remains mainly unchanged since its original publication in 2015, some notable bug fixes and improvements, such as the enhanced handling of rhombohedral space groups and disorder around special positions, have been introduced over the years. The full list of such changes is provided in the CHANGELOG file that is distributed with the cod-tools software package.

### Assignment of chemical structures

The next step in the pipeline deals with the assignment of bonds, bond orders, formal charges and other chemical features based on the atomic coordinates. This task is performed by the newly developed cif-perceive-chemistry [16] program which takes a stoichiometric CIF file as input and outputs a description of a chemical structure either as a structure-data file (SDF) [25] or as a tab-separated values (TSV) [26, 27] file that encodes the structure in a more compact form interpretable by the OpenChemLib framework [28]. The program can also be invoked with the “--tsv-output”, “--print-tsv-header” and “--print-dwar-header” options to prepend additional file headers which transform a TSV file into a native DataWarrior [29] (DWAR) file [30].

The core algorithm of cif-perceive-chemistry is similar to the one proposed by Roger Sayle for small-molecule ligand extraction from PDB files [4], however, in the presently reported approach the set of heuristics was specifically tailored to address the considerably greater chemical variety observed in small-molecule crystal structures. This resulted in an improved recognition of functional groups and aromatic systems as well as the introduction of special conventions to represent metal-coordination complexes (see the “Conventions” section). Furthermore, additional crystallographic information such as the attached hydrogen atom counts and crystal space group is also considered when determining the chemical structure. The current version of the program does not implement the processing of polymer descriptions, therefore any input files which are explicitly marked as containing polymeric molecular entities are rejected with an explicit error message.

The algorithm consists of the following steps:

1. Determination of atomic connectivity.
2. Assignment of unambiguous higher order bonds.
3. Assignment of unambiguous functional groups.
4. Assignment of aromatic and resonance structure bonds.
5. Assignment of higher order bonds that deviate from the expected geometry.
6. Assignment of formal charges.

The algorithm operates under the assumption that all hydrogen atoms in the input file are explicitly described either by providing their atomic coordinates, by using the _atom_site_attached_hydrogens data item or by employing a combination of both. The _atom_site_attached_hydrogens data item allows to record the number of hydrogen atoms that are known to be attached to a specific atom, but that were not reliably detected during the crystallographic experiment and are therefore not represented by a set of atomic coordinates. Consequently, processing of input files with insufficient hydrogen atom descriptions will always result in incorrect chemical structures, however, such entries are expected to be identified and excluded by external data quality filters described in the “Additional data validation criteria” section.

The cif-perceive-chemistry program aims to produce chemical structure descriptions that faithfully reflect the input crystal structures. Due to this, once the chemical structure is assigned no additional attempts are made to further convert it to some standard tautomeric or resonance form. For example, the azide functional group may be represented either as consisting of two double bonds or as consisting of a single bond and a triple bond depending on the actual reported interatomic bond lengths.

#### The interatomic bond length distribution set

Results of the cif-perceive-chemistry program heavily depend on the knowledge about typical bond lengths for any two connected atoms with a given bond order and a known neighbour environment. Histograms of bond length distributions for any characterised bond taken from experimental crystallographic data of similar bonds are used as one of the criteria when assigning the most plausible bond types and orders as well as validating the final chemical structure. The resulting dataset describes multiple bond classes each of which is approximated as a Gaussian distribution. The bond classes were constructed following an approach used by the OpenChemLib framework which considers bond properties such as the bond type and order as well as properties of the bonded atoms such as the atomic number and the $\pi$ electron count (see Additional file 1: Section S5). The bond length distribution set used in this work is available as part of the cif-perceive-chemistry software package as well as in a more human-readable form in Additional file 2.

#### Conventions

The same chemical structure can be represented in multiple different ways depending on the adopted conventions and the limitations of the data format. Some of the most popular chemical file formats (such as SDF V2000, SMILES and InChI) seem to be designed with the valence bond theory in mind, thus simplifying the link between atoms to two-centre bonds with precise bond orders and supposing that the charge of any moiety is localised on specific atoms. While this approach usually yields reasonable representations for organic compounds, it is insufficient for most species involving metal atoms such as metallocenes. As a result, researchers [14], organisations [13] and software developers [15] have to introduce their own conventions and assumptions in order to use the available formats. The cif-perceive-chemistry program also follows its own set of conventions that strive to be human-readable, convenient in data processing tasks and easily interconvertible with previously established approaches if needed. A summary of the more uncommon methods of structure representation is provided below.

***Aromatic systems.*** Aromatic systems are expressed using the Kekulé representation, i.e., as a set of interchanging single and double bonds. The resonance form is selected in a way that avoids introducing atoms with opposite formal charges and minimises the bond length deviations from the previously observed values. If the aromatic system has a delocalized charge as in the cases of the cyclopentadienyl ($[C_5H_5]^-$) and cyclooctatetraenide ($[C_8H_8]^{2-}$) anions, it is represented by placing a formal integer charge on one or more atoms in the system that have compatible valences.

***Metal atoms.*** Any atom with the atomic number from the set $[3,4] \cup [11,13] \cup [19,31] \cup [37,51] \cup [55,84] \cup [87,103]$ is considered a metal atom. This definition is inherited from the OpenChemLib framework and includes all atoms from the metal categories (alkali, alkaline earth, transition, lanthanide, actinide and post-transition metals) as well as Sb and Po atoms from the metalloid category.

***Zero-order coordination bonds.***

**Fig. 2:**
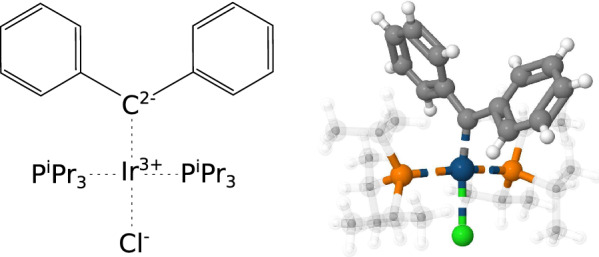
2D and 3D representations of a metal coordination complex that follow the conventions of the cif-perceive-chemistry program. Single and double covalent bonds are marked using solid lines while the zero-order coordination bonds are marked using dashed lines. In the 3D representation carbon and hydrogen atoms that belong to the triisopropylphosphine (P$^i$Pr$_3$) ligands are depicted in a translucent manner for legibility purposes. The double bond between the carbon and iridium atoms reported in the original publication [31] is expressed as a zero-order bond with an extra 2- formal charge on the carbon atom and a 2+ formal charge on the iridium atom. The 3D structure is based on COD entry 4076812 and was visualised using Jmol [32]

Current iteration of the algorithm does not attempt to resolve the most likely order of bonds involving metal atoms and instead marks all such bonds as coordination bonds with the bond order of 0. These bonds are ignored when assigning formal atomic charges and thus may lead to some atoms having seemingly unconventional charges (see Fig. 2). Zero-order coordination bonds are represented as “any bonds” (bond type 8; see [25], page 46) when using the MDL Molfile V2000 format and as “coordination bonds” (bond type 9; see [25], page 11) when using the MDL Molfile V3000 format. While the MDL Molfile V2000 format description explicitly states that bond types 4 through 8 should only be used for data queries, in practice this rule is often ignored and the bond range is used to represent certain additional features of the molecule. In applications that rely on the OpenChemLib framework, zero-order bonds are represented using the native MetalLigand bond type.

This simplified model is currently indiscriminately applied to all metal-containing entities including those which could be more accurately described by using only the conventional covalent bonds of various orders. This was a conscious choice motivated by the lack of a better automated solution rather than the limitations of the commonly used molecular representation formats. However, while in some cases the produced representations do not reflect the bonding typically used by chemists and may thus prove less useful in certain cheminformatics applications, the described approach still provides generalized insights into the bonding environments of various metal atoms. Furthermore, the assigned atomic charges and zero-order bonds should provide sufficient information for the users to normalize such metal-containing entities to the preferred more conventional representations. The creation of software that automates this task is planned as a future project and will greatly depend on the feedback received from the wider cheminformatics community.

**Fig. 3:**
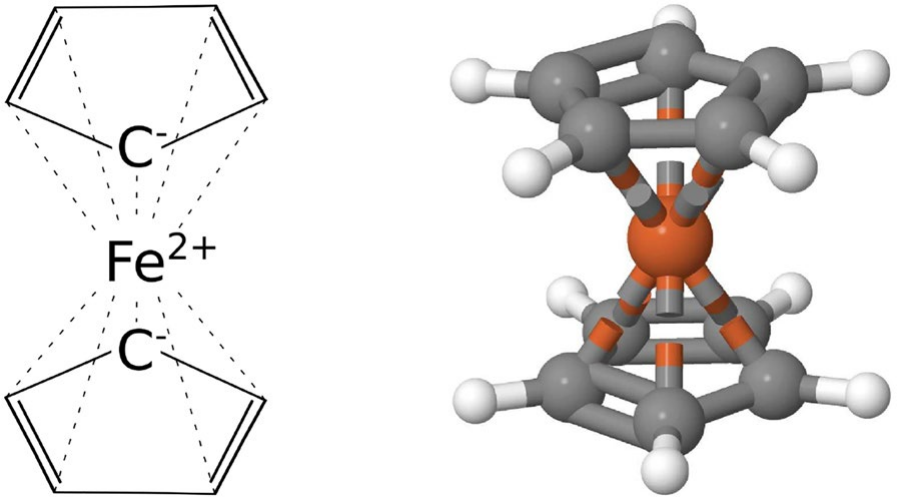
2D and 3D representations of a ferrocene molecular entity that follow the conventions of the cif-perceive-chemistry program. Single and double covalent bonds are marked using solid lines while the zero-order coordination bonds are marked using dashed lines. The 3D structure is based on COD entry 2101932 and was visualised using Jmol [32]

***Hapticity.*** Compounds in which a metal centre coordinates a ligand via an uninterrupted and contiguous series of atoms is represented by placing zero-order coordination bonds between the metal centre and all of the aforementioned coordinated atoms. For example, ferrocene is represented as an iron atom bound to all 10 carbon atoms of the two cyclopentadienyl molecules with one carbon atom from each ring being assigned a 1- charge and the iron atom being assigned a 2+ charge (see Fig. 3).

### Chemical structure validation

Before outputting a generated chemical structure, the cif-perceive-chemistry program validates it against a set of molecular geometry checks:

- $\pi$ **electron count.** Evaluates if the number of higher-order bonds that an atom is involved in is compatible with the molecular geometry of the atom. This is done by identifying the expected $\pi$ electron count values based strictly on the chemical species and the geometry of a given atom and comparing it with the factual $\pi$ electron count determined from the finalised structure. The factual atom $\pi$ electron count value is calculated by summing up the $\pi$ electron counts of each bond that the atom participates in with the assumption that a double bond requires a single $\pi$ electron, while a triple bond requires two $\pi$ electrons. Atoms which participate in at least one delocalized bond as defined in Additional file 1: Section S4 are assumed to contribute only a single $\pi$ electron. This test only applies to chemical elements B, C, N, O, Si, P, S, Cl, Ge, As, Se, Br, Te and I.
- **Bond type based on molecular geometry.** Evaluates if the assigned bond type is compatible with the bond angles and torsion angles formed by the bonded atoms and the neighbouring atoms. Bond lengths are not considered during this check.
- **Bond type based on previously observed bond lengths.** Evaluates if the bond length significantly differs from previously observed bond lengths of the same type between the same types of atoms. A bond length is considered to differ significantly if it falls outside the range of three standard deviations ($3\sigma$) from the mean and there is a bond type with a more compatible observed bond length distribution.
- **Radical atoms.** Evaluates if a molecular entity contains a radical atom.
- **Formal charge of atoms.** Evaluates if the formal charge assigned to an atom belongs to the predefined set of allowed charge values for the associated chemical element. The allowed charge values for metal atoms are determined based on the enumerated oxidation states while the allowed charge values for other atoms are determined based on their known valences as specified in the com.actelion.research.chem.Molecule.java class from the OpenChemLib framework version 2022.11.1 [33]. The same atom property information is also compiled in Additional file 3.
- **Formal charge of the stoichiometrically correct molecular ensemble.** Evaluates if the sum formal charge of atoms from all molecular entities is 0.

Any detected discrepancies are recorded in the COD_SDF_ISSUES data item of the SDF files (see Additional file 1: Section S6.3) and in the “C1-Problems” column of the DWAR file (see Additional file 1: Section S6.2).

### Additional data validation criteria

Each molecule description is also subjected to a set of data quality tests that determine if it is suitable for external use such as cross-linking with other databases. These tests consider not only the results of the chemical structure validation, but also the data discrepancies present in the input CIF file that may no longer be identifiable in the generated molecule description. For example, a CIF file that incorrectly contains only some of the atomic coordinates may still result in a valid chemical structure, just not the one that was originally reported in the associated peer-reviewed publication.

Data quality tests used in this work focus on the following characteristics:

- **Chemical structure.** Checks if the validation of the chemical structure using the cif-perceive-chemistry program did not report any discrepancies described in the “Chemical structure validation” section.
- **Chemical formula.** Checks if the chemical formula declared in the original CIF and the one calculated from the stoichiometric CIF are compatible. Formulae are considered compatible if they are identical or if the stoichiometric CIF formulae is a multiple of the declared chemical formula (e.g. “C12 H24 O12” and “C6 H12 O6”). The latter situation occurs in cases when the stoichiometric CIF files contain multiple instances of each molecular entity as described in the “Restoration of stoichiometrically correct molecular ensembles” section.
- **Data provenance.** Checks if sufficient bibliographic references to the original publication are provided.
- **Compositional disorder.** Checks if the original CIF file contains at least one site of compositional disorder that may lead to a suboptimal molecule description as discussed in the “Handling of crystallographically disordered structures” section.
- **COD entry markup.** Checks if the CIF entry was marked by the COD maintainers as unfit for calculations, i.e., as a duplicate, suboptimal or theoretical entry.
- **Manual review.** Checks if the chemical description was marked for exclusion by the COD maintainers after a manual review.

Entries that fail at least one validation criterion are explicitly marked as such in the SDF files using the COD_SDF_VALIDITY_STATUS data item and in the DWAR file using the “Is valid entry” column.

### Data representation, metadata, and available formats

In the final steps of the pipeline the molecule descriptions are expressed using several cheminformatics data formats. The successfully processed portion of the initial dataset is recorded as individual SDF files each of which represents a specific COD entry and consists of a single MDL Molfile V2000 compound description accompanied by several data items (see Additional file 1: Section S6.3). While the cif-perceive-chemistry program can also output results in the newer MDL Molfile V3000 format, the MDL Molfile V2000 format was chosen due to the greater compatibility with existing software and other databases (e.g. PubChem). In addition to that, the same data is also output as a single DWAR file (see Additional file 1: Section S6.2) with molecule descriptions encoded by the OpenChemLib framework as compact machine-readable text strings. An example DWAR file that describes a small subset of the COD is provided as Additional file 4. The DWAR file is readily interpretable by the DataWarrior program which can be used to carry out a great variety of chemical data analysis tasks or export the descriptions in multiple other cheminformatics data formats (see Additional file 1: Section S8). Finally, the SMILES, InChI and InChIKey linear representations are also produced.

The linear representations are generated from the SDF files using obabel from the Open Babel software package [34] version 3.0.0. Since chemistry perception is already performed by the cif-perceive-chemistry program and recorded fully in the SDF files, the call to obabel is supposed to only convert formats without modifying any of the chemical features. However, there is a slight problem with the handling of coordination bonds regardless of whether they are represented in the SDF file using the non-standard V2000 notation or the official V3000 notation (see the “Conventions” section). While during the conversion to SMILES obabel automatically changes coordination bonds to single bonds, conversion to InChI or InChIKey results in an “Unrecognized bond type” error message. This issue is addressed by converting SDF V2000 files to CML files using the same obabel program and then explicitly dealing with the coordination bonds. The initial conversion produces CML files in which the coordination bonds are represented as bonds with the bond order “8”. Then the XML elements specifying such bonds are either removed or reassigned the “1” bond order using the cml-manage-bonds program from the cml-tools software package version 0.2.0 [35]. Finally, the resulting CML files are once again piped into obabel to produce the desired linear representations.

These steps are used to generate multiple SMILES, InChI and InChIKey strings for each entry that describe either the entire crystal, the individual molecular components, or the individual ligands. When coordination bonds are represented as single bonds, representations are generated for the entire crystal and for the separate connected components using the obabel functionality enabled by the “--separate” option. The individual ligand representation is obtained by removing all coordination bonds and once again running obabel with the “--separate” option. This way, a separate string is obtained for each ligand as opposed to a single string that describes the entire metal-ligand complex.

All three representations have their own applications. Representation of the entire crystal allows to search for chemically identical crystals while the representation of individual molecular components allows to spot identical molecular entities such as coordination complexes in different crystals. The individual ligand representation that is obtained after the removal of coordination bonds allows to identify the same ligands in different coordination complexes. While all these functionalities could alternatively be achieved by running multiple chemical searches solely on the entire crystal representations, retaining all three sets of linear representations provides the option to interpret them as simple text strings which can be conveniently handled using a set of POSIX-compatible command line tools. In addition, this approach enables scientific data resources to efficiently integrate and link to the COD contents based on their use case, whether it be the individual components or the overall crystal structure or somewhere in-between.

Finally, it should be stressed that both SMILES and InChI currently provide quite limited standard means of describing metal-containing entities. For example, the SMILES specification does not define a coordination bond type nor does it allow to distinguish between all possible cases of coordination isomerism. Similarly, standard InChI identifiers do not contain the information required to distinguish between two coordination spheres involving the same ligands since all bonds to metal atoms are removed during the structure normalization stage [36]. As a workaround, one may choose to adopt additional SMILES conventions [13] or to generate non-standard InChI identifiers with the additional Reconnected Layer, however, this is not always feasible depending on the planned applications. Nevertheless, despite the aforementioned drawbacks both SMILES and InChI can still be used to efficiently identify candidate crystallographic structures that may describe the desired metal-containing entity while the final filtering can be delegated to a more resource-intensive method that analyses the 3D coordinates.

### Manual curation

A fully automated molecule derivation pipeline has some clear benefits when compared to a more manual approach, however, it also comes with some limitations. On the one hand, an automated approach is much faster, has better scalability and generates results that are more uniform and easier to reproduce. On the other hand, it is hard to develop a system that is as sophisticated as a trained specialist and does not easily make errors when presented with partially incorrect data or a novel type of compound. Due to this, the pipeline described in this work takes an intermediate approach and automates straightforward tasks while also relying on human expertise to resolve the more complex issues or provide additional hints to the system.

Manual curation efforts in the COD pipeline are directed at several stages. Part of the effort is dedicated to correcting issues in the original COD CIF files such as incorrectly marked atom disorder or unmarked attached hydrogen atoms. Another curation task deals with cases where the default way of generating stoichiometrically correct molecular ensembles as described in the “Restoration of stoichiometrically correct molecular ensembles” section is not sufficient even though the input CIF file is completely valid. Such situations require the creation of specialised per-entry configuration files which adjust the behaviour of the cif_molecule program, for example, by changing the maximum bond length cutoff parameter that influences the assumed interatomic connectivity. Finally, curators have the option to explicitly mark certain suspicious entries as invalid based on their expertise. Currently, the data curator pool is limited to the COD maintainers, however, with enough interest the system could be updated to enable the participation of the wider scientific community.

Entries identified as invalid according to the criteria described in the “Additional data validation criteria” section do not automatically get excluded from the distributed dataset but are rather explicitly marked using appropriate data fields. While it is generally desirable to have a dataset that is as issue-free as possible, it could also be argued that providing all available data with clearly marked problems may be preferred under some circumstances since it allows the end user to make an informed choice on whether certain entries should be retained based on the planned application. This is extremely important in the context of the described pipeline since automated checks may sometimes incorrectly mark a sound molecular description as having issues just because it deviates from previously observed molecular geometries (see the “Chemical structure validation” section). However, when exchanging data with external databases any manually or automatically flagged COD entries are automatically excluded to avoid the propagation of even potentially incorrect data.

## Results

The chemical description derivation pipeline was used on all 473 500 CIFs from revision 265250 of the COD. Chemical descriptions were successfully generated for about 68% of all entries, with calculations for the remaining entries terminating at various stages of the pipeline for various reasons as summarised in Table 1:

- <1% (683 of 473 500) could not be properly processed by the cif_molecule program either due to exhaustion of assigned computational resources or inherent problems in the input files such as atoms with unrecognised chemical symbols, missing atomic coordinates, insufficient space group information, etc. A more detailed summary of the encountered issues is provided in Additional file 1: Section S9.
- <1% (4 313 of 473 500) could not be properly processed by the cif-perceive-chemistry program due to the exhaustion of assigned computational resources or the limitations imposed by the used data format. That is, MDL Molfile V2000 format is not suited to describe molecules with more than 999 atoms or 999 bonds in a single file.
- 8% (23 668 of 473 500) were preemptively excluded from further processing by the cif-perceive-chemistry program after detecting certain characteristics that are guaranteed to result in an incorrect chemical description. For example, an atom that has more bonded neighbours than is allowed by its maximum valence or a steric clash between two atoms will always result in unreasonable bond distances or charges and is usually a consequence of incorrectly marked disorder in the original input CIF.
- 23% (106 620 of 473 500) were recognised as describing polymers which are currently not supported by the cif-perceive-chemistry program. In the scope of this work a polymer is defined as a molecular entity which consists of periodically repeating identical subunits and spans an infinite number of crystal unit cells.

**Table 1:** Summary of the issues that prevented COD CIFs from being successfully processed in the chemical description derivation pipeline. The three rows in bold describe the part of the dataset that was successfully processed, the part of the dataset that was excluded from further calculations, and the overall input dataset

Status	Entry count	% of all entries
**Successfully processed**	**322 776**	**68.17**
**Excluded due to one of the following reasons:**	**150 724**	**31.83**
*Describes polymers*	106 622	22.52
*Contains steric clashes (“atomic bumps”)*	23 668	5.00
*Contains atoms with exceeded valency*	15 438	3.26
*Fails when processed by* *cif_molecule*	4 313	0.91
*Exceeds MDL Molfile V2000 limitations*	680	0.14
*Exceeds allocated CPU time*	2	0.00
*Raises uncategorised errors*	1	0.00
**Total**	**473 500**	**100.00**

All generated molecule descriptions, were then tested against a set of additional criteria to evaluate their suitability for cross-linking with other curated resources as described in the “Additional data validation criteria” section. Out of 322 776 descriptions about 73% were identified as completely valid while the rest failed at least one data quality check. A summary of the most common validation issues is provided in Table 2. Although not evident from the summary table, there is a quite high correlation between the issue categories with the most affected entries: 9% of all descriptions contain only chemical structure issues, 8% contain only chemical formula issues, while 6% contain both.

**Table 2:** Summary of the chemical description validation results. Since the same description may manifest issues from more than one category, the total number of invalid entries is less than the sum of entries from all issue categories. The three rows in bold describe the part of the dataset that successfully passed all validation checks, the part of the dataset that failed at least one validation check, and the overall dataset used in the validation

Validation status	Entry count	% of all entries
**Successfully validated**	**236 328**	**73.22**
**Failed one or more checks related to:**	**86 448**	**26.78**
*Chemical structure*	57 504	17.82
*Chemical formula*	46 307	14.35
*Compositional disorder*	8 314	2.58
*Data provenance*	4 653	1.44
*COD entry markup*	3 084	0.96
*Manual review*	406	0.13
**Total**	**322 776**	**100.00**

All successfully generated SDF files as well as the complete DWAR file were made available under the CC0 licence and can be retrieved using several different methods described in Additional file 1: Section S7. This dataset includes all entries regardless of their determined validity status, thus additional entry filtering may be needed depending on the planned usage. Entries that failed at least one data validity criteria can be easily identified by checking the value of the COD_SDF_VALIDITY_STATUS SDF file data item (see Additional file 1: Section S6.3) or the “Is valid entry” DWAR file column (see Additional file 1: Section S6.2). Chemical descriptions that raised no validation issues were uploaded to PubChem under the “Crystallography Open Database” data source [37] (see the “COD data integration into PubChem” section).

## Discussion

The molecule derivation pipeline described in this work was purposely designed to only use open-source software to enable its reuse and to benefit from the insights of the wider scientific community. Similarly, the open nature of the COD enabled the distribution of the derived data under the permissive CC0 licence which places it into the public domain with no additional restrictions. It has already been demonstrated that the molecule descriptions generated by this pipeline are extremely useful in tasks related to the stewardship of the COD data such as the detection of data inconsistencies [38] and the cross-linking with external resources. Furthermore, the derived dataset provides a convenient way to carry out data search queries in the COD that simultaneously involve both the chemical and the crystallographic data such as polymorph detection. Finally, the data can also be used outside of the COD for purposes such as machine learning or the derivation of molecular geometry parameter sets.

The current version of the pipeline is capable of successfully addressing the large variety of crystallographic structures observed in the COD, however, there remain several open issues related to the representation of certain crystallographic concepts using data formats popular in cheminformatics. Among these issues is the handling of crystallographically disordered structures.

### Handling of crystallographically disordered structures

Crystallographic disorder describes a physical phenomenon when different cells of the same crystal differ in chemical composition or spatial arrangements of atoms. The two most common disorder types are positional disorder and compositional disorder, although a combination of the two is also possible. Positional disorder occurs when the same set of atoms have slightly different positions in different unit cells while compositional disorder describes situations when the same atom site in different unit cells is occupied by different types of atoms. There is also a relatively common special case of compositional disorder called partial occupancy which occurs when a chemical species is present only in a fraction of equivalent crystallographic positions across different unit cells. While the CIF framework provides the means to describe atom disorder in several different ways, most of the widely used cheminformatics formats (e.g., SMILES, SDF) do not explicitly specify how such compounds should be represented.

Positional disorder can be handled quite reasonably by generating a representative conformation as described in Additional file 1: Section S2, however, this heuristics-based approach may still sometimes produce a suboptimal combination of disordered atom positions. Take, for example, COD entry 7109837 which contains disorder groups A:1 and A:2 that both consist of the same number of atoms with identical occupancies thus leading to the selection of group A:1 that has a lexicographically lesser name. However, due to the way the disordered site was modelled, atoms from group A:1 have steric clashes while atoms from group A:2 constitute a tetrafluoroborate anion with a reasonable conformation. A non-exhaustive search in the COD identified only 14 additional entries [39] that result in similar issues, though, a more in-depth investigation is required to determine the prevalence of such crystal structures and the overall fitness of the representative conformation selection algorithm proposed in this work. Alternatively, one may generate all possible combinations of disordered positions, process them using a chemical comprehension program and, choose the best out of the valid ones. Analysis of the COD revealed that, out of 69 561 entries that only contain positional disorder, 90% are limited to no more than four distinct combinations thus making this a feasible complementary approach.

Compositional disorder, on the other hand, poses a more serious challenge. For example, COD entry 7014723 describes a crystal of a metal complex with a compositionally disordered metal atom site that is occupied either by an iron or a cobalt atom with the ratio of 1:1. When generating a chemical description of such crystal structures each variant of the metal complex together with any counter ions or solvent molecules may be represented as separate entries, keeping in mind that this approach does not properly relay the observed chemical composition. Alternatively, all possible variants of the metal complex may be included in the same entry while preserving the correct stoichiometry, however, this approach becomes unfeasible with structures that have a greater number of positionally disordered sites or sites with greater occupancy ratios (e.g., COD entry 2231340 contains a metal atom site with a ratio of 1:49). Due to this, the chemical perception pipeline described in this work takes a conservative approach and completely excludes structures which contain compositional disorder sites occupied by multiple chemical species instead of misrepresenting them.

Finally, partially occupied atom sites are treated in the same way as atom sites with full occupancy. While this approach over-represents the partially occupied molecular entities in the chemical description, it may still provide useful insights into the molecular composition of the disordered crystals. These chemical descriptions, however, are also automatically excluded when cross-linking with other databases based on criteria such as unbalanced charges or mismatching chemical formulae as described in the “Additional data validation criteria” section. 

### COD data integration into PubChem

**Fig. 4:**
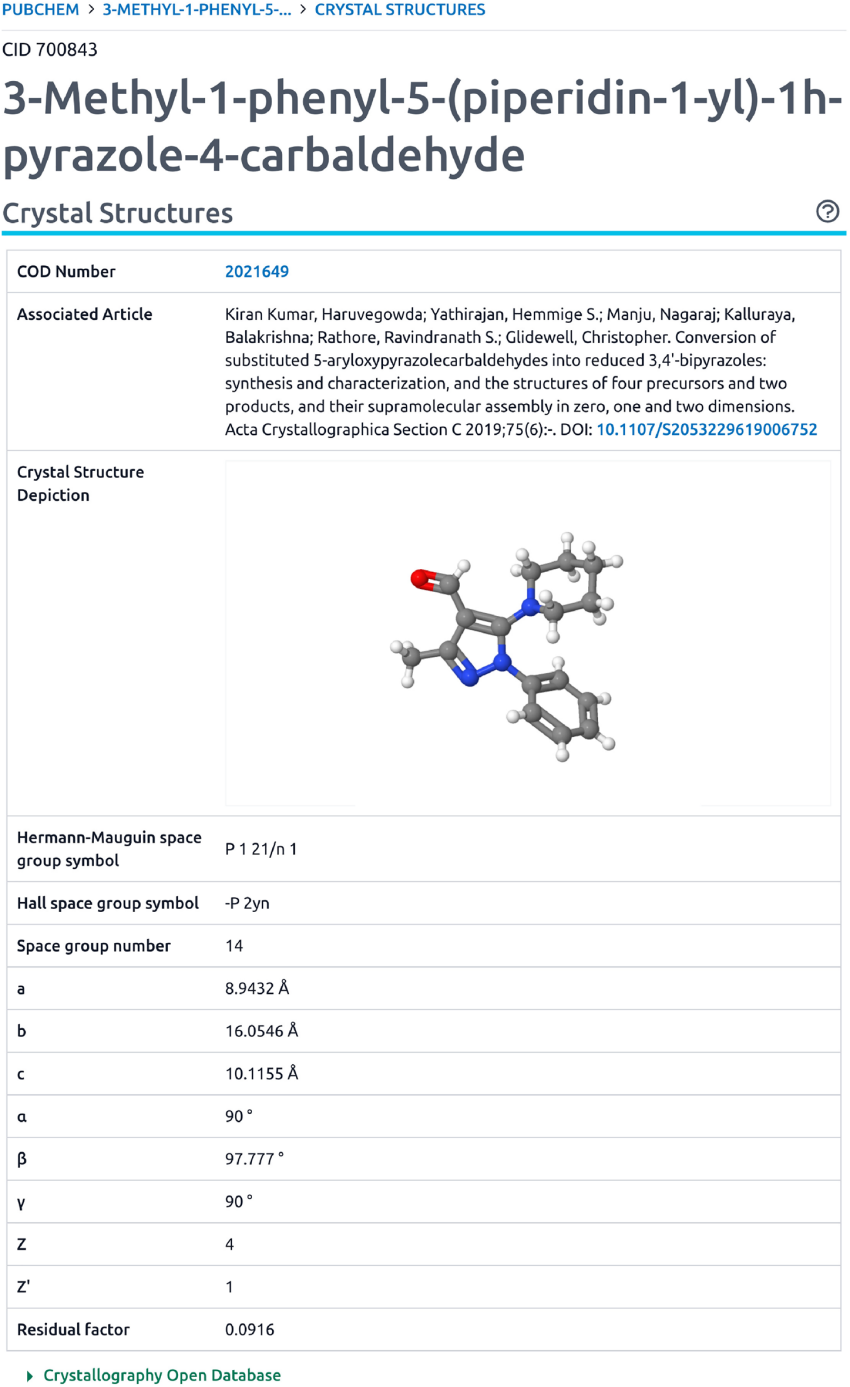
Depiction of Crystal Structure information from COD in PubChem for CID 700842 [40]

**Fig. 5:**
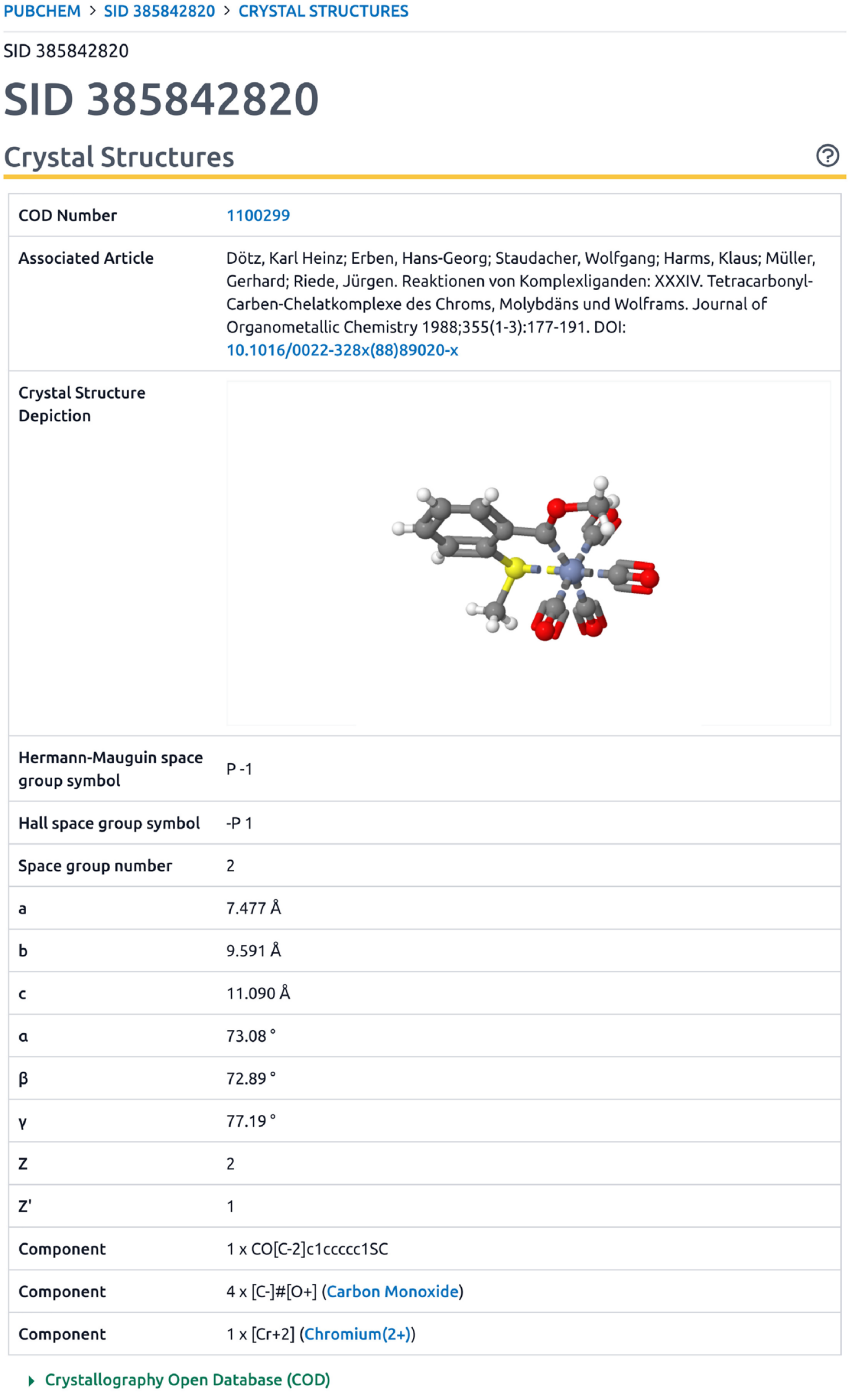
Depiction of Crystal Structure information from COD in PubChem for SID 385842820 [41]

Great care was taken to integrate COD records in PubChem in several ways. The COD first became a PubChem Substance data contributor in 2013 with the initial deposition of a few hundred records. PubChem does not directly accept crystal structure formats, which need to be converted into a chemical structure format like SMILES to be accepted in PubChem. This current work substantially expands upon earlier efforts to convert crystal structure data into chemical structure formats and now covers nearly the entirety of non-polymeric COD entries, which, as of July 2023, contains 245 473 records in PubChem Substance. Also of great importance, is that earlier data in PubChem included only chemical structure data with links back to the COD. Now, specific information about the corresponding crystal structure records is provided to and integrated by PubChem. This includes a depiction, COD identifier, DOI of the crystal structure publication, publication citation information, space group symbol (Hermann-Mauguin and Hall), space group number, unit cell parameters (a, b, c, $\alpha$, $\beta$, $\gamma$), Z, Z’, and residual factor. This is exemplified by Fig. 4 depicting, as an example, CID 700843 (*3-Methyl-1-phenyl-5-(piperidin-1-yl)-1 h-pyrazole-4-carbaldehyde*). Given that not all crystal structure data readily converts into valences expected by small molecule resources like PubChem, and therefore not always assigned a compound identifier, this annotation is also provided for COD substance records in PubChem as shown in Fig. 5 for SID 385842820. Beyond annotation content, PubChem also indicates cases when component compounds of a mixture have crystal structure information. This data integration is automated. As such, regular monthly updates enable new content to be continually provided as the COD resource expands.

## Conclusion

In this work we presented a computational pipeline capable of deriving molecular descriptions from small-molecule crystallographic data with minimal human intervention. The stoichiometrically correct molecular ensemble restoration step is performed by a previously published cif_molecule program [21] while the chemical perception step is carried out by a newly developed cif-perceive-chemistry program that aims to make greater use of available crystallographic data and introduces an alternative way of describing metal coordination complexes. In the final stages of the pipeline the results are evaluated against a set of data quality criteria that cover both the generated molecular descriptions and the original crystallographic files (Additional file 4).

The pipeline was successfully used to create an open-access collection of molecular descriptions from the crystallographic data of the COD. All the generated results were made available for download in various cheminformatics data formats. Entries that passed all of the data quality criteria were uploaded to PubChem as a set of SDF files with experimentally determined 3D coordinates. This served as the first large scale attempt to link PubChem with the COD and showcased the validity of the approach for bridging chemical and crystallographic data.

### Scientific contribution

In this work, we have developed a computational pipeline capable of deducing 3-dimensional chemical structures from small-molecule crystallographic models and applied it to the crystal structures in the Crystallography Open Database (COD). This resulted in an open-access collection of chemical entities with experimentally observed 3D coordinates which was successfully used to cross-link crystallographic data in the COD with chemical information in the PubChem database. The presented software is completely open-source and thus may be applied to crystallographic datasets other than the COD while the derived chemical entity dataset may be useful in applications that simultaneously require both chemical and crystallographic data.

### Supplementary Information

**Additional file 1: ** PDF file that describes the methods and data formats used in this works in greater detail, and provides instructions on how the produced dataset can be retrieved.

**Additional file 2: ** Tab-separated value file that contains descriptions of bond length distribution classes used in this work. A detailed description of each column is provided in Additional file 1: Section S5.

**Additional file 3: **YAML file that contains the atom properties such as known oxidation states and known formal charges. A more detailed description of the serialised data structure is provided in the human-readable comment at the start of the file.

**Additional file 4: **DWAR file that has the same structure as a regular COD DWAR file, but only describes the 4 crystal structures that were explicitly referenced in the article instead of the entire COD dataset. A detailed description of a COD DWAR file is provided in Additional file 1: Section S6.2.

## Data Availability

The input CIF files are distributed under the CC0 license and can be retrieved from the Crystallography Open Database (COD), https://www.crystallography.net/cod. The calculated chemical structure dataset in SDF and DWAR formats is distributed under the CC0 license and can be retrieved from https://molecules.crystallography.net. The calculated chemical structure dataset was also uploaded to PubChem under the “Crystallography Open Database (COD)” data source https://pubchem.ncbi.nlm.nih.gov/source/849.

## Code availability

The cif-perceive-chemistry software package is released under the GNU GPLv3 license and can be obtained free-of-charge from the svn://www.crystallography.net/cif-perceive-chemistry Subversion repository. The work described in this paper was carried out using version 0.4.0 of the cif-perceive-chemistry software package which can be retrieved from https://doi.org/10.5281/zenodo.7510339. The cod-tools software package is released under the GNU LGPLv3 license and can be obtained free-of-charge from the svn://www.crystallography.net/cod-tools Subversion repository. The work described in this paper was carried out using version 3.6.0 of the cod-tools software package which can be retrieved from https://www.crystallography.net/cod/archives/2022/software/cod-tools/cod-tools-3.6.0.tar.gz. The cml-tools software package is released under the MIT license and can be obtained free-of-charge from svn://saulius-grazulis.lt/cml-tools Subversion repository. The work described in this paper was carried out using version 0.2.0 of the cml-tools software package which can be retrieved from svn://saulius-grazulis.lt/cml-tools/tags/v0.2.0.
