# Supplementary material for: A workflow for deriving chemical entities from crystallographic data and its application to the Crystallography Open Database
Source: J Cheminform. 2023 Dec 19;15:123. doi: 10.1186/s13321-023-00780-2 (PMC10730636; doi:10.1186/s13321-023-00780-2)
Supplement: Supplementary file 1 — Additional file 1: PDF file that describes the methods and data formats used in this works in greater detail, and provides instructions on how the produced dataset can be retrieved. [file 13321_2023_780_MOESM1_ESM.pdf]

A workflow for deriving chemical entities from crystallographic data and its application to the Crystallography Open Database

Supplementary material

Antanas Vaitkus[1*], Andrius Merkys[1], Thomas Sander[2], Miguel Quirós[3], Paul A. Thiessen[4], Evan E. Bolton[4] & Saulius Gražulis[1,5]

[1] Section of Crystallography and Chemical Informatics, Institute of Biotechnology, Life Sciences Center, Vilnius University, Saulėtekio al. 7, LT-10257, Vilnius, Lithuania
[2] Scientific Computing Drug Discovery, Idorsia Pharmaceuticals Ltd, Hegenheimermattweg 89, 4123 Allschwil, Switzerland
[3] Departamento de Química Inorgánica, Universidad de Granada, 18071, Granada, Spain
[4] National Center for Biotechnology Information, National Library of Medicine, National Institutes of Health, Bethesda, MD 20894, USA
[5] Faculty of Mathematics and Informatics, Vilnius University, Naugarduko g. 24, LT-03225, Vilnius, Lithuania
[*] *E-mail: antanas.vaitkus@bti.vu.lt*

October 2, 2023

Contents

S1 Additional COD resources used for entry validity determination 1

S2 Selection of a representative conformation of a disordered molecule 1

S3 Derivation of the interatomic bond length distribution set 1

S4 On aromatic and delocalized bonds 2

S5 Description of the bond length distribution set 2

S6 Available data formats 3
- S6.1 Stoichiometric CIF file 3
- S6.2 DWAR file 3
- S6.3 SDF files 6

S7 Data retrieval instructions 7
- S7.1 Retrieval of SDF files 7
- S7.2 Retrieval of the DWAR file 7

S8 Example of using the COD DWAR file with the `DataWarrior` program 8

S9 Overview of fatal errors that arose while processing COD CIFs 11

S1 Additional COD resources used for entry validity determination

Chemical descriptions generated from COD entries must satisfy certain quality criteria to be suitable for cross-linking with external resources. Only entries that pass all of the data quality tests are marked as valid and are allowed to remain in the final dataset. Some of the tests require the following additional summary files and local databases:

- `cif-formulae-mismatch.tsv`. A tab-separated value file which lists COD entries for which chemical formula declared in the original input CIF file differs from the chemical formula calculated from the corresponding stoichiometric CIF file. Such mismatches may be used to detect discrepancies in the input crystal structures such as missing atoms, incorrectly marked crystal symmetry or incorrect chemical formulae.
- `COD.sqlite3`. An `SQLite` database which contains various properties of the input crystal structures such as cell constants, chemical formulae, experimental conditions, data provenance information as well as descriptions introduced by the COD database maintainers (e.g. the duplicate structure flag).
- `disorder-in-cod.sqlite3`. An `SQLite` database which contains information about explicitly marked disorder in the input crystal structures.

These resources are generated from the same fixed COD revision as the one used to produce the final chemical descriptions.

S2 Selection of a representative conformation of a disordered molecule

The `cif_molecule` program selects a representative conformation of a molecule in a disordered crystal structure by identifying the optimal combination of disordered group positions based on the following rules of decreasing priority:

- Groups with higher occupancies are preferred. In the scope of this algorithm an entire disordered group is assigned the highest occupancy that was observed among its constituent atom sites.
- Groups with a higher number of disordered atom sites are preferred.
- Groups with a lexicographically lesser name (unique identifier) are preferred.

The described approach produces satisfactory results with most positionally disordered structures but does not comprehensively represent compositionally disordered structures (see the "Handling of crystallographically disordered structures" section of the main publication).

S3 Derivation of the interatomic bond length distribution set

The interatomic bond length distribution set used by the `cif-perceive-chemistry` program was derived from open data using the following algorithm:

1. Calculate chemical structures of all COD entries using the `Open Babel` software suite by converting the stoichiometric CIF files to SDF files.
2. Determine the set of trustworthy chemical structures. A chemical structure is considered trustworthy if it matches the structure represented by a SMILES string from an expert-curated COD SMILES dataset [1]. The comparison of chemical structures is carried out by converting both structures to canonical SMILES and comparing them as case sensitive text strings.
3. Derive the bond length distribution dataset based on the set of trustworthy chemical structures identified in step 2.
4. Recalculate chemical structures of all COD entries this time using the `cif-perceive-chemistry` program with the newly derived bond length distribution dataset.
5. Determine the set of trustworthy chemical structures. This time a chemical structure is considered trustworthy if it does not contain significant deviations from previously observed molecular geometry and does not contain obvious errors such as an unbalanced molecular charge.

6. Derive the bond length distribution dataset based on the set of trustworthy chemical structures identified in step 5.

Sequential versions of the bond lengths distribution set (e.g., derived from future revisions of the COD database that may contain more entries) can be calculated by repeating steps 4–6.

S4 On aromatic and delocalized bonds

The data model of a molecule used by the `OpenChemLib` framework includes calculated atom and bond properties that may not have been explicitly specified in the input data and were instead determined using specific rules. Bond aromaticity and bond delocalization are two such properties that are used by the `cif-perceive-chemistry` program when assigning and validating a chemical structure. A bond is considered *aromatic* if it is in a ring and the ring satisfies the Hückel's rule. Similarly, a bond is considered *delocalized* if it is aromatic and if the ring does not have a preferred mesomeric state. For example, all bonds in a pyridine ring are recognised as delocalized while all bonds in a thiophene ring are recognised as aromatic, but not as delocalized. SDF files produced by the chemical perception pipeline do not explicitly identify aromatic or delocalized bonds in any way while the DWAR file retains this information using machine-readable *idcode* text strings that encode molecular structures in a canonical and compact way.

S5 Description of the bond length distribution set

Bonds in the bond length distribution set used by `OpenChemLib` and `cif-perceive-chemistry` are classified using the following bond properties:

- **Bond order**. A formally assigned enumeration state which classifies the bond as a single, double, or triple bond.
- **Bond aromaticity**. A flag value which signifies if the bond was recognised as aromatic based on the rules specified in Section S4.
- **Bond delocalization**. A flag value which signifies if the bond was recognised as delocalized based on the rules specified in Section S4.
- **Atomic numbers of the bonded atoms**. The atomic numbers of the bonded atoms as specified in the periodic table of chemical elements.
- **π electron counts of the bonded atoms**. The overall π electron count of an atom is calculated by summing up the π electron counts of each bond that an atom participates in with the assumption that a double bond requires a single π electron, while a triple bond requires two π electrons. Atoms which participate in at least one delocalized bond are assumed to contribute only a single π electron. The π electron count is only considered when dealing with chemical elements B, C, N, O, P and S.

Additional file 2 contains the bond length distribution set used in this work expressed in a tab-separated value format with the following columns:

- `bond_id`. A unique identifier of the bond class that also encodes the bond properties in a compact way.
- `bond_length`. The mean of the bond length distribution of the given bond class in ångströms.
- `bond_std`. The standard deviation of the bond length distribution of the given bond class in ångströms.
- `bond_count`. The number of bond length observations used to calculate the distribution.
- `bond_type`. An alphanumeric string that identifies a combination of bond order and bond flag values. Values "1", "2" and "3" denote a single, double, and triple covalent bond. Values "a1" and "a2" denote single and double aromatic bond, respectively. Value "d" denotes a delocalized bond.
- `atom_1_symbol`. The chemical element symbol of bond atom `a1`.
- `atom_2_symbol`. The chemical element symbol of bond atom `a2`.

- atom_1_pi_count. The π electron count of bond atom a1. This number is set to 0 if the chemical element is not one of B, C, N, O, P or S.

- atom_2_pi_count. The π electron count of bond atom a2. This number is set to 0 if the chemical element is not one of B, C, N, O, P or S.

The same information in the cif-perceive-chemistry software package is encoded as a machine-readable resources/bondLengthData.txt file that is directly interpretable by the OpenChemLib framework. Note, however, that since a custom approach was used to derive the bond length distributions (see Section S3), values provided in this file differ from those given in the default bond length distribution set file distributed as part of OpenChemLib.

S6 Available data formats

S6.1 Stoichiometric CIF file

In the scope of this work a *stoichiometric CIF file* is defined as a CIF file which explicitly lists all atoms of a stoichiometrically correct molecular ensemble instead of providing the conventional crystal structure description that lists atoms from the asymmetric unit. This type of file is usually generated from a conventional CIF file using the cif_molecule program with the "--one-datablock-output", "--preserve-stoichiometry", "--largest-molecule-only" and "--split-disorder-groups" command line options.

A stoichiometric CIF file fully conforms to the CIF file syntax, but reuses some of the data items from the CIF_CORE dictionary [2] with slightly different semantics. The main differences include:

- The ATOM_SITE category loop explicitly describes all atoms that make up the molecule instead of only the asymmetric unit atoms.

- Data items dealing with the space group information (e.g., _space_group_symop_operation_xyz, _space_group_name_Hall) always describe the P 1 space group rather than the space group of the input crystal structure. Since the P 1 space group consists of a single x,y,z identity operation, no additional symmetrically-equivalent atoms are generated even if the symmetry operations are reapplied to the atoms described in the ATOM_SITE category loop.

- The _cell_formula_units_Z data item is always set to "1".

While this type of informal redefinition of the semantics may be viewed as a drawback, the reuse of existing data items makes the files readily interpretable by various external pieces of software such as Jmol or obabel. Furthermore, there are ongoing IUCr discussions [3] on the introduction of the _audit.formalism data item that would allow to describe such reuse cases in a more explicit way. Finally, files produced by the cif_molecule program can normally be reliably identified by examining the contents of the _audit_creation_method data item.

A stoichiometric CIF file may also contain a set of COD data items that record various additional properties such as the identified presence of a polymeric molecule (_cod_molecule_is_polymer) or the original space group of the input crystal structure (_cod_molecule_space_group_IT_number). Definitions of these data items are provided in the CIF_COD [4] and CIF_COD_MOLECULE [5] dictionaries.

S6.2 DWAR file

COD molecule descriptions are also distributed as a single DWAR file [6]. The DWAR file consists of an XML-like header that is intended to be interpreted by the open-source DataWarrior program followed by a data table. The data table adheres to the formatting rules of tab-separated value file and consists of a header row followed by multiple data rows, each of which describes a separate COD entry. An example DWAR file that has the same structure as a regular COD DWAR file, but only describes the 4 crystal structures that were explicitly referenced in the main publication instead of the entire COD dataset is provided as Additional file 4.

DWAR files distributed by the COD contain the following data columns:

- Structure. A machine-readable *idcode* text string that encodes the molecular structure in a canonical and compact way. Generated by and intended to be interpreted by the OpenChemLib framework.

- idcoordinates3D. A machine-readable *idcoordinates3D* text string that encodes the 3D atomic coordinates of the molecular structure provided in the Structure field. Generated by and intended to be interpreted by the OpenChemLib framework.

- `FragFp`. A machine-readable text string that encodes the *FragFp* binary fingerprint of the molecular structure provided in the `Structure` field. Relies on a dictionary of 512 predefined structure fragments. Generated by and intended to be interpreted by the `OpenChemLib` framework. For more information, see the "Similarity & Descriptors" section of the `DataWarrior` user manual [7].

- `SkelSpheres`. A machine-readable text string that encodes the *SkelSpheres* descriptor of the molecular structure provided in the `Structure` field. Generated by and intended to be interpreted by the `OpenChemLib` framework. For more information, see the "Similarity & Descriptors" section of the `DataWarrior` user manual [7].

- `Source file`. The name of the original COD CIF file that was used to generate the molecule description. Derived from the value of the `_cod_data_source_file` data item provided in the input CIF file. Used for data provenance purposes.

- `Source block`. The code of the data block within the original COD CIF file that was used to generate the molecule description. Derived from the value of the `_cod_data_source_block` data item provided in the input CIF file. Used for data provenance purposes.

- `Has attached hydrogen atoms`. A "`yes`"/"`no`" flag value which indicates if any of the atoms in the input CIF were marked as having attached hydrogen atoms instead of providing explicit coordinates of those hydrogen atoms. Note that molecule descriptions generated from CIF files marked with the "`yes`" value will contain at least some hydrogen atoms without explicit 3D coordinates. For more information on the concept of attached hydrogen atoms, see the description of the `_atom_site_attached_hydrogens` data item from the `CIF_CORE` dictionary.

- `Space group IT number`. The number of the space group that the molecule crystallised in. This number is derived from symmetry information provided in the input crystallographic file and follows the conventions described in the International Tables for Crystallography, Volume A. Among other applications, the space group number can be used to identify whether certain chiral molecular entitites represent a single enantiomer or a racemate in the processed crystal structure as discussed in the "Restoration of stoichiometrically correct molecular ensembles" section of the main publication.

- `Software package name`. The name of the program that generated the molecule description. Used for data provenance purposes.

- `Software package version`. The version string of the program that generated the molecule description. Used for data provenance purposes.

- `Creation timestamp`. The molecule description creation timestamp in ISO 8601 format. Used for data provenance purposes.

- `C1-Problems`. A summary of discrepancies detected by the chemical structure validation tests described in the "Chemical structure validation" section of the main publication. The validation results are recorded as a single line where individual issues are separated by a single space symbol and each issue is expressed as a compact ASCII text string that follows an internal shorthand notation. An empty string indicates that no issues were detected.

- `COD Number`. A persistent unique identifier of the original input COD entry. Also known as a COD ID.

- `Substance Name`. The trivial name by which the compound is commonly known as. Derived from the value of the `_chemical_name_common` data item provided in the input CIF file.

- `Chemical Name`. IUPAC or Chemical Abstracts full name of the compound. Derived from the value of the `_chemical_name_systematic` data item provided in the input CIF file.

- `Authors`. A list of authors of the peer-reviewed publication that describes the crystal structure. Derived from the value of the `_publ_author_name` data item provided in the input CIF file. The used name syntax follows the BibTeX convention which is slightly different from the convention described by the IUCr in the definition of the `_publ_author_name` data item provided in the input CIF file.

- `Title`. The title of the peer-reviewed publication that describes the crystal structure. Derived from the value of the `_publ_section_title` data item provided in the input CIF file.

- `Journal`. The name of the journal in which the peer-reviewed publication that describes the crystal structure was published in. Derived from the value of the `_journal_name_full` data item provided in the input CIF file.
- `Year`. The publication year of the peer-reviewed publication that describes the crystal structure. Derived from the value of the `_journal_year` data item provided in the input CIF file.
- `Volume`. The journal volume of the peer-reviewed publication that describes the crystal structure. Derived from the value of the `_journal_volume` data item provided in the input CIF file.
- `Issue`. The journal issue of the peer-reviewed publication that describes the crystal structure. Derived from the value of the `_journal_issue` data item provided in the input CIF file.
- `First Page`. The first page of the peer-reviewed publication that describes the crystal structure. Derived from the value of the `_journal_page_first` data item provided in the input CIF file.
- `Last Page`. The last page of the peer-reviewed publication that describes the crystal structure. Derived from the value of the `_journal_page_last` data item provided in the input CIF file.
- `DOI`. The DOI of the peer-reviewed publication that describes the crystal structure. Derived from the value of the `_journal_paper_doi` data item provided in the input CIF file.
- `Method`. The method which was used to determine the crystal structure as identified using a set of heuristics. Takes one of the values from the [“`single crystal`”,“`powder diffraction`”,“`theoretical`”] enumerated set. For more information on how this value is determined see the description of the “`method`” field in the COD SQL database description [8].
- `Radiation`. The type of radiation which was used to determine the crystal structure. Derived from the value of the `_diffrn_radiation_probe` data item provided in the input CIF file.
- `Wavelength`. The wavelength of the radiation which was used to determine the crystal structure in ångströms. Derived from the value of the `_diffrn_radiation_wavelength` data item provided in the input CIF file.
- `R-factor all`. The residual factor for all reflections satisfying the resolution limits. For more information on how this value is determined see the description of the “`Rall`” field in the COD SQL database description [8].
- `Cell length a`. The lattice parameter a of the crystal structure in ångströms. Derived from the value of the `_cell_length_a` data item provided in the input CIF file.
- `Cell length b`. The lattice parameter b of the crystal structure in ångströms. Derived from the value of the `_cell_length_b` data item provided in the input CIF file.
- `Cell length c`. The lattice parameter c of the crystal structure in ångströms. Derived from the value of the `_cell_length_c` data item provided in the input CIF file.
- `Cell angle alpha`. The lattice parameter *alpha* of the crystal structure in degrees of arc. Derived from the value of the `_cell_angle_alpha` data item provided in the input CIF file.
- `Cell angle beta`. The lattice parameter *beta* of the crystal structure in degrees of arc. Derived from the value of the `_cell_angle_beta` data item provided in the input CIF file.
- `Cell angle gamma`. The lattice parameter *gamma* of the crystal structure in degrees of arc. Derived from the value of the `_cell_angle_gamma` data item provided in the input CIF file.
- `Cell volume`. The volume of the crystal lattice calculated from the lattice parameters in cubic ångströms.
- `Space group H-M`. The space group symbol of the crystal structure as described by Hermann-Mauguin. May be replaced by a superspace group symbol if one is explicitly defined in the input CIF file. For more information on how this value is determined see the description of the “`sg`” field in the COD SQL database description [8].
- `Space group Hall`. The space group symbol of the crystal structure as described by Hall. Derived from the value of the `_space_group_name_Hall` data item provided in the input CIF file.
- `Is valid entry`. A “`yes`”/“`no`” flag value which indicates if an entry successfully passed all of the COD data quality tests.

S6.3 SDF files

The following data items may appear in the SDF files [9] distributed by the COD:

- `COD_SDF_CIF_SVN_REVISION`. Revision number of the input CIF file in the COD Subversion repository.
- `COD_SDF_DATA_SOURCE_FILE`. Name of the input CIF file.
- `COD_SDF_DATA_SOURCE_BLOCK`. Name of the CIF data block from the input CIF file.
- `COD_SDF_CREATION_TIMESTAMP`. Timestamp recorded at the end of the SDF file creation.
- `COD_SDF_SOFTWARE_PACKAGE_NAME`. Name of the software package that was used to create the SDF file.
- `COD_SDF_SOFTWARE_PACKAGE_VERSION`. Version of the software package that was used to create the SDF file.
- `COD_SDF_SPACE_GROUP_IT_NUMBER`. The number of the space group that the molecule crystallised in. This number is derived from symmetry information provided in the input crystallographic file and follows the conventions described in the International Tables for Crystallography, Volume A [10]. Among other applications, the space group number can be used to identify whether certain chiral molecular entitites represent a single enantiomer or a racemate in the processed crystal structure as discussed in the "Restoration of stoichiometrically correct molecular ensembles" section of the main publication.
- `COD_SDF_STRUCTURE_HAS_ATTACHED_HYDROGENS`. A flag value that indicates if any of the atoms in the input CIF were marked as having attached hydrogen atoms instead of providing explicit coordinates of those hydrogen atoms. Enumeration values:
  - `yes`. The input CIF file was marked as having attached hydrogen atoms therefore some hydrogen atoms will not be represented as distinct atoms in the SDF file.
  - `no`. The input CIF file was not marked as having attached hydrogen atoms therefore all hydrogen atoms will be represented as distinct atoms in the SDF file. This is the default value and thus often omitted.
- `COD_SDF_ATTACHED_HYDROGEN_ATOMS`. A multiline list that records the number of attached hydrogen atoms that were assigned to specific atoms. Each line in the list consists of an atom index and a corresponding number of attached hydrogen atoms separated by a single space symbol.
- `COD_SDF_ISSUES`. A summary of discrepancies detected by the chemical structure validation tests described in the "Chemical structure validation" section of the main publication. The validation results are recorded as a single line where individual issues are separated by a single space symbol and each issue is expressed as a compact ASCII text string that follows an internal shorthand notation. An empty line indicates that no issues were detected.
- `COD_SDF_VALIDITY_STATUS`. A flag value that indicates if the original COD entry adheres to a set of additional quality criteria such as the presence of sufficient bibliographic information, match between the generated and calculated chemical formulae, etc. Enumeration values:
  - `1`. Entry successfully passed all the quality criteria tests.
  - `0`. Entry failed at least one of the quality criteria tests or the generated molecule description contains some deviations from the expected results (see the COD_SDF_ISSUES data item).
- `PUBCHEM_EXT_DATASOURCE_REGID`. Unique identifier of the input COD entry (COD ID).
- `PUBCHEM_EXT_DATASOURCE_URL`. URL of the COD website landing page (`https://www.crystallography.net/cod/`).
- `PUBCHEM_EXT_SUBSTANCE_URL`. URL of the input COD entry.
- `PUBCHEM_SUBSTANCE_COMMENT`. Bibliographic reference to the original publication that describes the input crystal structure.
- `PUBCHEM_SUBSTANCE_SYNONYM`. Chemical name of the substance observed in the crystal as extracted from the `_chemical_name_systematic` and `_chemical_name_common` CIF data items of the input CIF file. May contain several alternative names, one name per line.

PubChem data items are used with the permission of PubChem maintainers and do not deviate from the original intent expressed in the PubChem documentation [11, 12].

S7 Data retrieval instructions

The generated molecular descriptions are distributed under the CC0 licence and can be retrieved in several ways. Data retrieval examples provided in this section were tested using a `Bash` shell under the Ubuntu 22.04 GNU/Linux system. For alternative ways to engage with these data, please visit `https://molecules.crystallography.net`.

S7.1 Retrieval of SDF files

The SDF files are organised in a way that is very similar to the way CIF files are organised in the main COD repository. Each SDF file is assigned a filename which corresponds to the 7-digit COD ID of the input COD entry and is placed in a directory tree location determined from the first 5 digits of the COD ID. For example, SDF file generated from COD entry 2231955 is assigned the `2231955.sdf` filename and placed in the `2/23/19/` directory. The described SDF file layout is used by the following endpoints that can be used to retrieve the data:

- `rsync://molecules.crystallography.net/sdf`. Accessible using the `rsync` protocol. This is the recommended method for downloading and updating the data when the entire SDF dataset is required. Retrieval of individual files using this method is also possible.
- `https://molecules.crystallography.net/cod/sdf`. Accessible using the `https` protocol, e.g. via `curl`, `wget` or a general purpose web browser such as Firefox.

Usage examples:

- Download SDF entry 2231955 using `rsync`:

```
rsync -avz rsync://molecules.crystallography.net/sdf/2/23/19/2231955.sdf 2231955.sdf
```

- Download the entire SDF dataset using `rsync`:

```
rsync -avz rsync://molecules.crystallography.net/sdf/ sdf
```

- Download SDF entry 2231955 using `curl` via the `https` endpoint:

```
curl https://molecules.crystallography.net/cod/sdf/2/23/19/2231955.sdf > 2231955.sdf
```

S7.2 Retrieval of the DWAR file

The dataset of all successfully processed COD entries is also distributed as a single DWAR file (see Section S6.2). The DWAR file can be retrieved from the following endpoints:

- `rsync://molecules.crystallography.net/dwar`. Accessible using the `rsync` protocol.
- `https://molecules.crystallography.net/cod/dwar/COD.dwar`. Accessible using the `https` protocol, e.g. via `curl` or `wget`. Accessing this endpoint using a general purpose web browser such as Firefox should be done with caution since some browsers may try to display the entire ≈500 MB file instead of initiating a file download.

Usage examples:

- Download the directory with the COD.dwar file using `rsync`:

```
rsync -avz rsync://molecules.crystallography.net/dwar/ dwar
```

- Download the DWAR file using `curl` via the `https` endpoint:

```
curl https://molecules.crystallography.net/cod/dwar/COD.dwar > COD.dwar
```

S8 Example of using the COD DWAR file with the DataWarrior program

The open-source DataWarrior program offers a large variety of functionalities for the analysis of chemical data. This section provides an example of how the DWAR file generated from the COD crystallographic data by the described workflow can be used for the detection of polymorphs. The provided example was tested on the Ubuntu 20.04 GNU/Linux system using the v05.05.00 version of DataWarrior downloaded directly from the developers website (https://openmolecules.org/datawarrior/download.html). Note, DataWarrior installers for Windows and MacOS-X systems are also available.

Steps to search the COD database for polymorphs of *sulfamerazine*:

1. **Download and install DataWarrior as specified in https://openmolecules.org/datawarrior/download.html.**

2. **Download the COD DWAR data file following instructions provided in Section S7.2 and rename it to COD.dwar if needed.**

3. **Start DataWarrior and open the COD.dwar file.** Click File → Open in the top toolbar and select the file. This should open a window similar to the one displayed in Figure S1. Note that the DataWarrior window is highly customisable thus undesired panels may be closed while the more relevant one may be moved or resized.

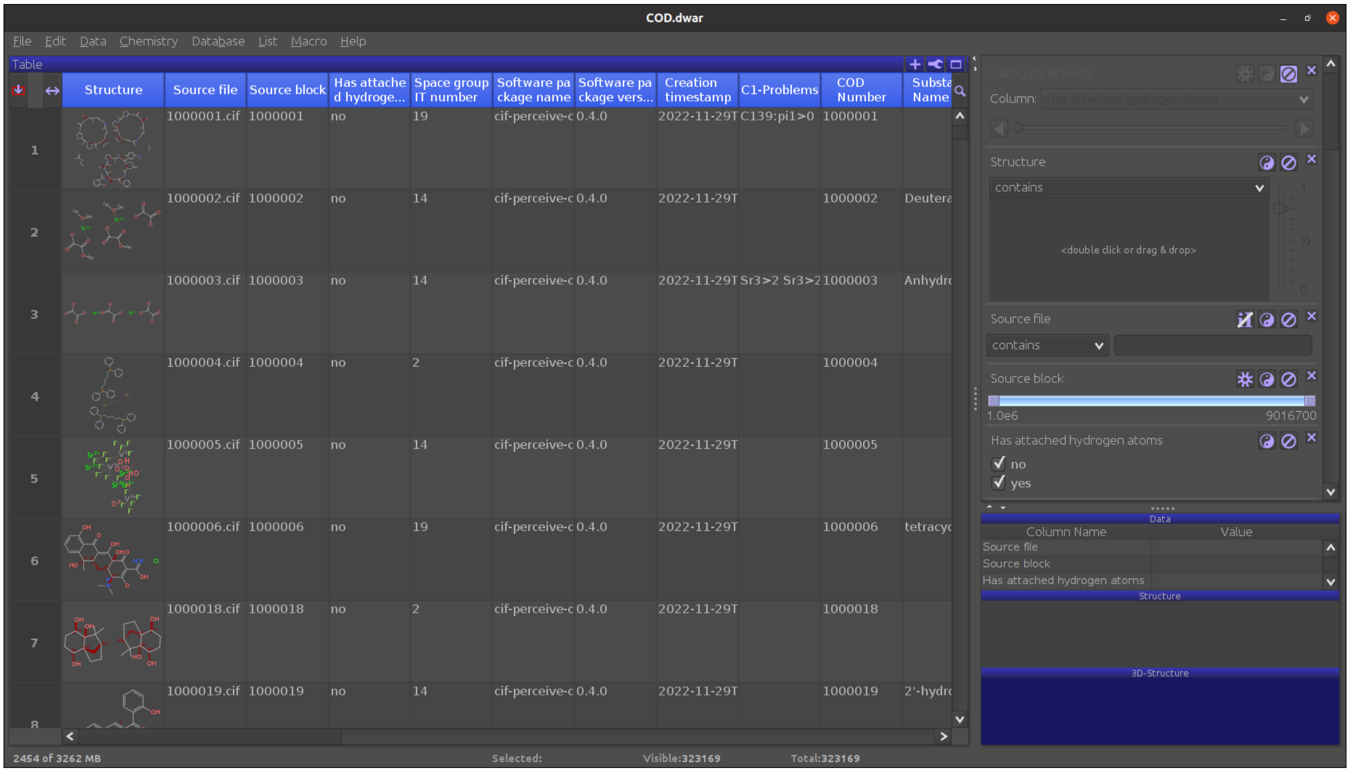

Figure S1: DataWarrior window upon the initial loading of the COD.dwar file.

4. **Apply search filters to exclude entries with undesired features.** By default, all of the automatically generated filters will be located in the scrollable panel on the right side of the screen (see Figure S2). The names of the filters correspond to the names of the data table columns to which they apply (see Section S6.2). Exclude all theoretical crystal structures by locating the Method section and removing the check mark from the Theoretical field. Exclude all structures that did not pass all of the COD data quality checks by locating the Is valid field and removing the check mark from the No field.

5. **Apply structure filter to select entries that contain only the compound of interest (*sulfamerazine*).** Scroll back to the top of the filter panel and locate Structure filter section. Copy the following

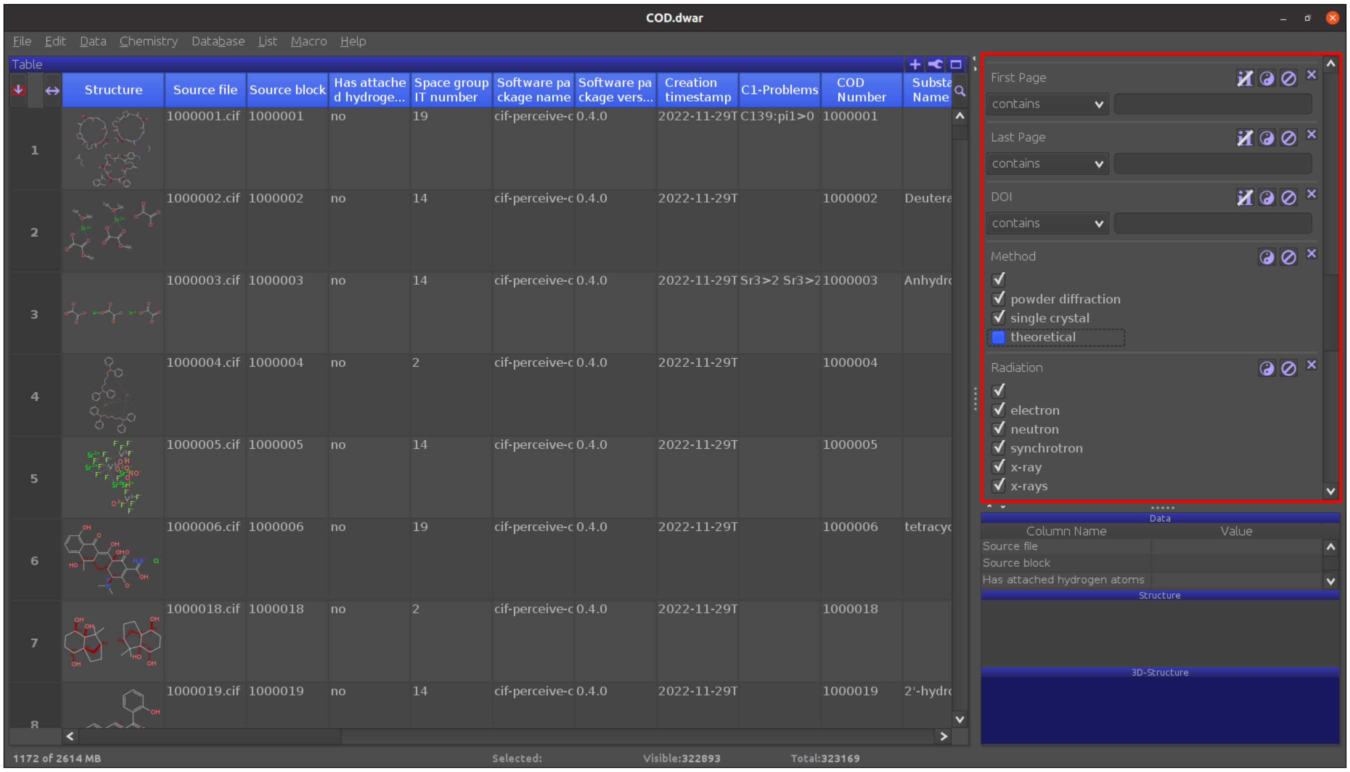

Figure S2: DataWarrior window after filtering out all marked theoretical structures. The scrollable panel that contains the filter fields is marked using red lines.

SMILES string "Cc1ccnc(NS(c(cc2)ccc2N)(=O)=O)n1", right click on the part of the Structure filter that reads "<double click or drag & drop>" and then click "Paste Structure or Name". This should draw the structural formula of *sulfamerazine* in the search field and automatically filter out all entries that do not fit this filter criterion (see Figure S3). Note, that the structure search field can be populated by multiple alternative methods (e.g. pasting a MDL Molfile, manually drawing the chemical fragment).

6. **Further refine the structure search to match only *sulfamerazine* and not its derivatives or similar molecular entities.** In the structure search field click on the "Is similar to [FragFP]" field and change it to "Is similar to [SkelSpheres]". Locate a slider on the left side of the chemical structure that goes from 0 to 1 and move it all the way up to 1 (see Figure S4).

7. **Click on the Space Group IT number column name to sort the remaining fields by the space group number.**

8. **Inspect the remaining data using the method of your choice to locate polymorphs, for example:**

   (a) Manually locate entries that have different space group numbers (recorded in the Space Group IT number column) or entries that have the same space group number, but significantly different cell lattice parameters (recorder in the a, b, c, alpha, beta, gamma columns).

   (b) Alternatively, automate the final analysis by exporting the selected entries into a separate TSV or SDF file and post-process them using custom external programs. Create a new subset by clicking File → New From → Visible rows in the top toolbar. This should open a new DataWarrior window that can be manipulated independently from the original one. In the newly created window click File → Save Special and select either Textfile... or SD-File....

Caveats of the described method:

- The list of selected structures may also include some racemic crystal structures. However, these can be easily recognised and filtered out using by the value of the Space Group IT number column (see column description in Section S6.2).

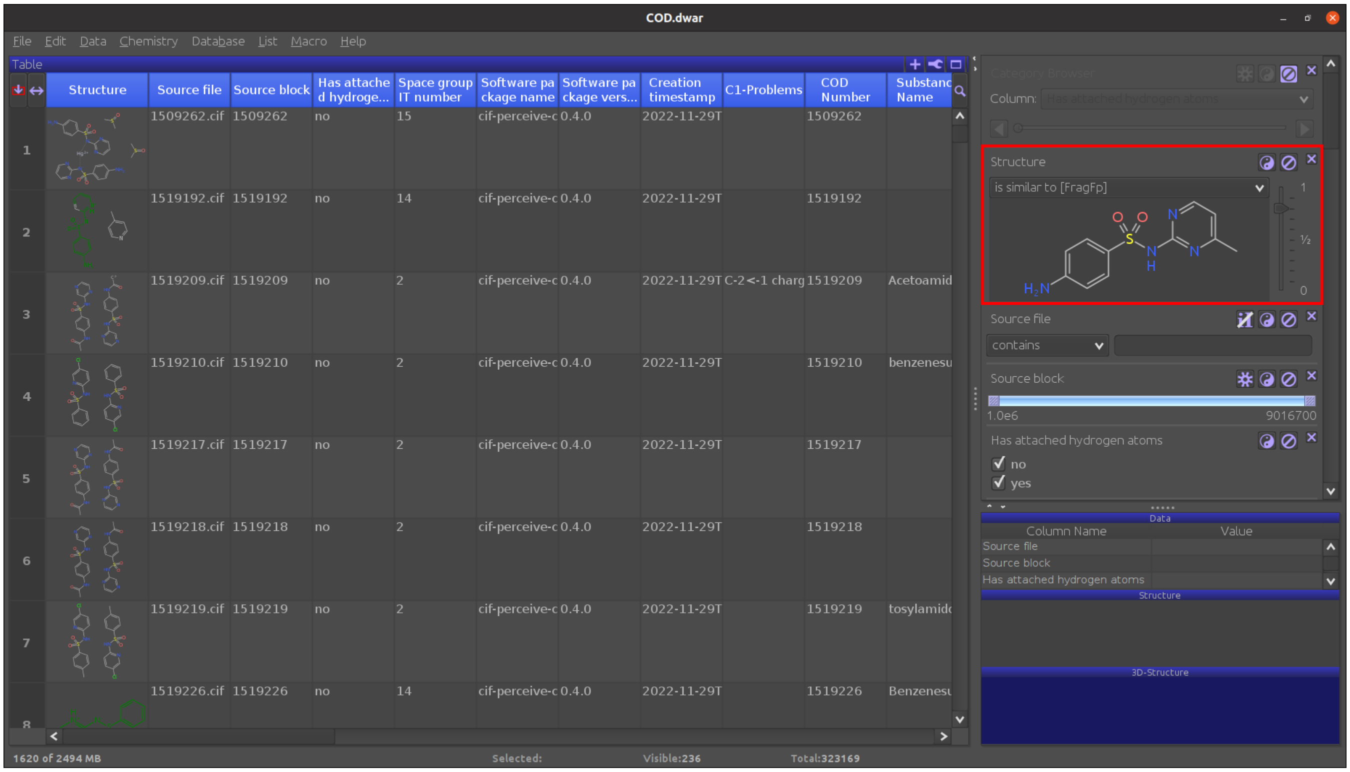

Figure S3: `DataWarrior` window after inputting the SMILES string of *sulfamerazine* into the structure search field. The structure search filed is marked using red lines.

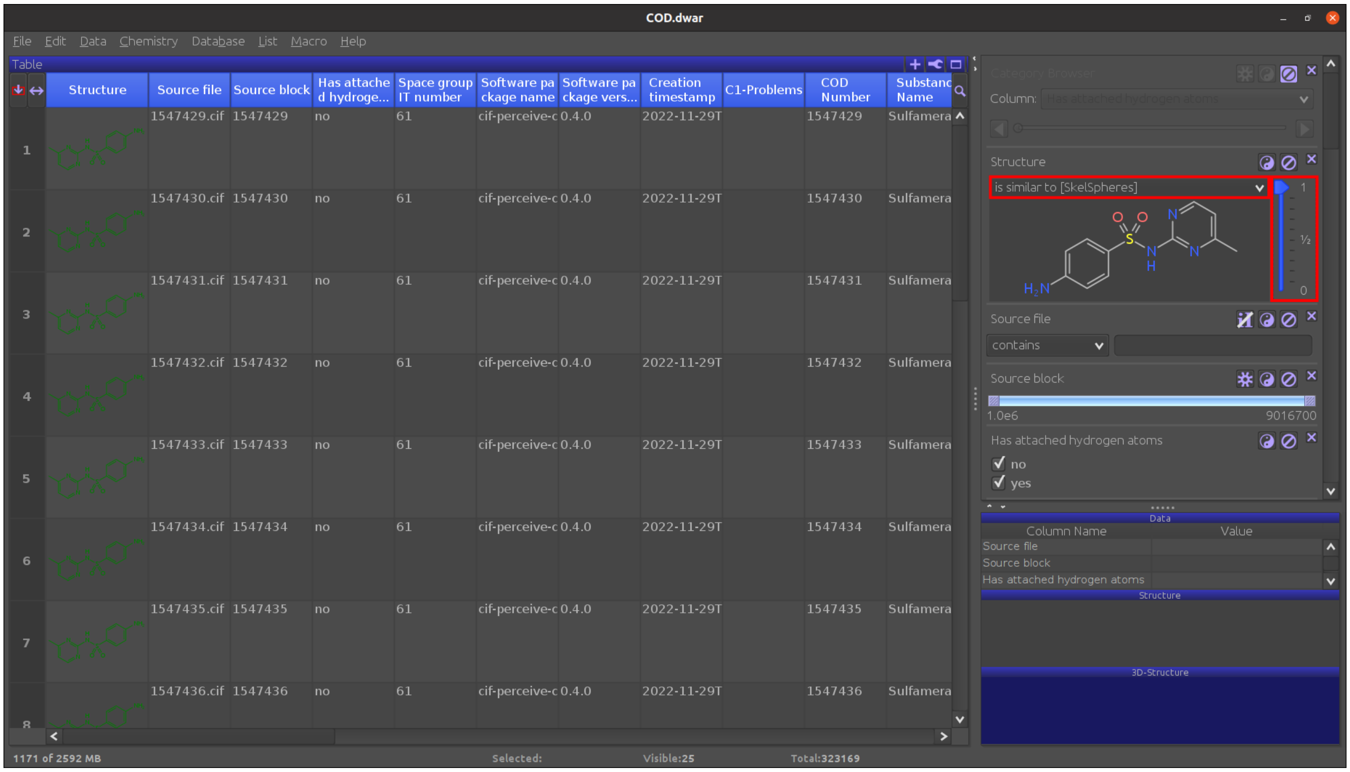

Figure S4: `DataWarrior` window after further adjusting the structure search parameters. The similarity type selection field and the similarity slider are marked using red lines.

- The list of selected structures may omit some legitimate crystal structures that consist entirely of sulfamerazine. This would only happen if in the input crystal structure the asymmetric unit contained more than one instance of a sulfamerazine entity. With some additional filtering steps this could be overcome.

S9 Overview of fatal errors that arose while processing COD CIFs

The chemical description derivation pipeline was used on all 473 500 CIFs from revision 265250 of the COD. Less than 1% of all CIFs could not be properly processed by the `cif_molecule` program due to various reasons that are summarised in Table S1.

**Error type**	**Entry count**	**% of all entries**
Unrecognised chemical type	2733	0.5772
No atomic coordinates	1220	0.2577
Insufficient symmetry information	327	0.0691
Max CPU time exceeded	17	0.0036
Out of memory	10	0.0021
Incorrect symmetry information	5	0.0011
No fractional coordinates	1	0.0002

Table S1: Summary of fatal errors that arose while processing COD CIFs with the `cif_molecule` program.

References

[1] M. Quirós, S. Gražulis, S. Girdzijauskaitė, A. Merkys, and A. Vaitkus, "Using SMILES strings for the description of chemical connectivity in the Crystallography Open Database", *Journal of Cheminformatics*, vol. 10, no. 1, May 2018. [Online]. Available: https://doi.org/10.1186/s13321-018-0279-6

[2] (2023) IUCr Core CIF development repository. IUCr. Last accessed: 2023-08-21. [Online]. Available: https://github.com/COMCIFS/cif_core/

[3] (2020) A guide to using formalisms and schemas in CIF. IUCr. Last accessed: 2023-08-21. [Online]. Available: https://github.com/COMCIFS/comcifs.github.io/blob/0f8ac3d5179959218fe39085a70685528dd3821a/draft/using_formalism_and_schema.md

[4] A. Vaitkus, A. Merkys, and S. Gražulis. (2020) CIF_COD DDL1 dictionary, version 0.050. COD. Last accessed: 2023-08-21. [Online]. Available: https://www.crystallography.net/cod/cif/dictionaries/ddl1/cif_cod/cif_cod_0.050.dic

[5] A. Vaitkus, A. Merkys, and S. Gražulis. (2022) CIF_COD_MOLECULE DDL1 dictionary, version 0.001. IUCr. Last accessed: 2023-08-21. [Online]. Available: https://www.crystallography.net/cod/cif/dictionaries/ddl1/cif_cod_molecule/cif_cod_molecule_0.001.dic

[6] T. Sander. (2023) The .dwar file format. [Online]. Available: https://openmolecules.org/help/fileformats.html

[7] T. Sander. (2023) Similarity & descriptors. Last accessed: 2023-08-21. [Online]. Available: https://openmolecules.org/help/similarity.html

[8] S. Gražulis, A. Merkys, and A. Vaitkus. (2022) COD SQL database description, version 1.2.0. COD. Last accessed: 2023-08-21. [Online]. Available: https://www.crystallography.net/cod/xml/documents/database-description/database-description_v1.2.0.xml

[9] "CTFile formats", BIOVIA, Tech. Rep., 2020, last accessed: 2023-08-21. [Online]. Available: https://discover.3ds.com/sites/default/files/2020-08/biovia_ctfileformats_2020.pdf

[10] M. I. Aroyo, Ed., *International Tables for Crystallography.* International Union of Crystallography, 2006, vol. A. [Online]. Available: https://doi.org/10.1107/97809553602060000114

[11] (2022) Substance tag names. PubChem. Last accessed: 2023-08-21. [Online]. Available: https://pubchemdocs.ncbi.nlm.nih.gov/substance-tag-names

[12] (2021) Substance tag names. PubChem. Archival link. Last accessed: 2023-08-21. [Online]. Available: https://web.archive.org/web/20221006045325/https://pubchemdocs.ncbi.nlm.nih.gov/substance-tag-names

[13] (2023) Crystallography Open Database - PubChem data source. PubChem. Last accessed: 2023-08-21. [Online]. Available: https://pubchem.ncbi.nlm.nih.gov/source/849
